# Diagnostic Ambiguity in BCR–ABL1–Positive MPAL in a Resource‐Limited Setting: A Case Report

**DOI:** 10.1155/crh/2170200

**Published:** 2026-09-22

**Authors:** Sadichhya Poudel, Subidha Sharma, Shankar Kafle, Shovana Karki, Anjan Shrestha

**Affiliations:** ^1^ Department of Pathology, Tribhuvan University Teaching Hospital/Maharajgunj Medical Campus, Kathmandu, Nepal

**Keywords:** BCR–ABL1, diagnostic challenge, leukemia, mixed phenotype, tyrosine kinase inhibitors

## Abstract

Mixed phenotype acute leukemia (MPAL) is a rare hematologic malignancy characterized by the presence of both myeloid and lymphoid lineage markers, posing significant diagnostic and therapeutic challenges. The presence of BCR–ABL1 further complicates classification and management, as it carries important prognostic and therapeutic implications. We report a case of BCR–ABL1–positive MPAL diagnosed through integrated morphological, immunophenotypic, and cytogenetic evaluation. The case highlights key diagnostic pitfalls and underscores the importance of comprehensive immunophenotyping and molecular testing to avoid misclassification as acute myeloid leukemia or acute lymphoblastic leukemia. Recognition of this entity is critical for appropriate therapeutic decision‐making, including the use of tyrosine kinase inhibitors. This report emphasizes the need for heightened awareness of MPAL with BCR–ABL1, representing a form of high‐risk acute leukemia with significant therapeutic implications, particularly in resource‐limited settings where diagnostic constraints may delay optimal management.

**Summary**
• Mixed phenotype acute leukemia (MPAL) is a rare hematologic malignancy with significant diagnostic and therapeutic challenges.
• This case of BCR–ABL1–positive MPAL, diagnosed through integrated morphological, immunophenotypic, and cytogenetic evaluation, emphasizes the need for heightened awareness of high‐risk acute leukemia with significant therapeutic implications, particularly in resource‐limited settings.

## 1. Introduction

Leukemia is a blood cancer resulting from uncontrolled proliferation of immature white blood cells; it is classified as acute or chronic (based on growth rate) and myeloid or lymphoid (based on cell of origin) [1]. Acute leukemia of ambiguous lineage (ALAL) is a heterogeneous group, revealing no specific lineage or with multiple lineages. Cases without lineage are acute undifferentiated leukemia (AUL), and those with multiple lineage markers are mixed phenotype acute leukemia (MPAL). MPALs are rare (< 5% of acute leukemia) and have a poorer prognosis than typical acute lymphoblastic leukemia (ALL) or acute myeloid leukemia (AML) [2]. Although classified as MPAL, BCR–ABL1–positive cases share important biological and therapeutic features with high‐risk AML, including aggressive clinical behavior and the need for intensified, targeted treatment strategies.

Throughout history, MPAL has undergone numerous modifications to its name and diagnostic standards [3]. The WHO 5th edition classifies ALAL by genetic abnormalities (BCR–ABL1, KMT2A, ZNF384, and BCL11B) and immunophenotype (B/Myeloid or T/myeloid), with cases not meeting the criteria designated as ALAL, NOS, including AUL [4].

In MPAL, lineage is assigned as myeloid by MPO positivity or ≥ 2 monocytic markers (CD11c, CD14, CD64, and lysozyme); T‐lineage by cytoplasmic or surface CD3; and B‐lineage by CD19 with supportive markers (CD79a, cCD22, or CD10) [5].

The BCR: ABL1 fusion arises from the Philadelphia chromosome translocation t (9; 22) (q34; q11), occurring in < 0.5% of AML cases and 15%–20% of MPAL cases [6]. Most MPAL patients are adult males, with BCR–ABL1 being the most common cytogenetic abnormality [7]. The fifth edition of the World Health Organization classification of hematolymphoid tumors (WHO‐HAEM5) classifies de novo MPAL with BCR–ABL1 (MPAL BCR–ABL1) as a clinical entity under the acute leukemia of mixed or ambiguous lineage grouping [4]. An immunophenotype that fulfills the diagnostic criteria for MPAL, BCR: ABL1, and/or t (9; 22) (q34; q11.2) detected at initial diagnosis, no prior or subsequent evidence of CML, and no history of exposure to cytotoxic therapy are vital diagnostic criteria for MPAL BCR–ABL1 patients. It is necessary to demonstrate that leukemic cells meeting criteria for B‐, T‐, or myeloid leukemia express markers associated with multiple hematopoietic lineages [4, 6, 8]. Patients with MPAL BCR–ABL1 often present with high WBC counts, anemia, thrombocytopenia, and splenomegaly or hepatomegaly and show fewer chromosomal abnormalities than AML BCR–ABL1. Poorer survival is associated with being older than 15 years, high WBC count, and the BCR–ABL1 fusion. The use of TKIs with ALL‐like induction has improved outcomes, and stem cell transplantation is recommended for treatment‐resistant cases [6, 8]. Here, we report a case of BCR–ABL1–positive MPAL diagnosed through integrated morphological, immunophenotypic, and cytogenetic evaluation.

## 2. Case Presentation

**FIGURE 1:**
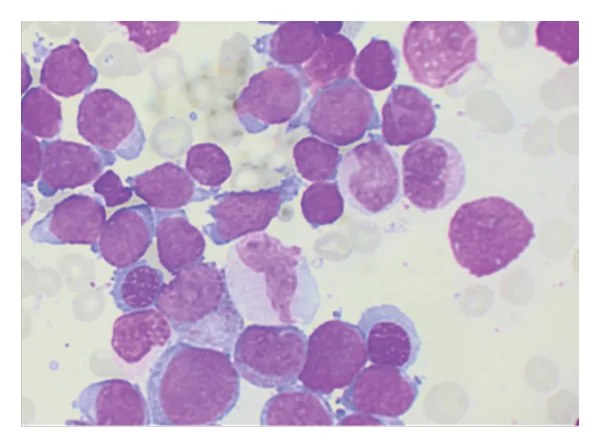
Bone marrow aspirate showing the presence of blasts (100x magnification). The blasts are of intermediate size, having a high N:C ratio, a moderate amount of dense basophilic agranular cytoplasm, round nuclei, fine chromatin, and prominent nucleoli. Cytoplasmic blebbing is also seen.

A 39‐year‐old woman with no prior medical history presented with two months of abdominal pain and exertional dyspnea, accompanied by fever, anorexia, vomiting, and weight loss. Physical examination revealed no lymphadenopathy or hepatosplenomegaly. Laboratory tests showed mild anemia (Hb 9.5 g/dL), leukocytosis (WBC 110,200/cumm), and thrombocytopenia (80,000/cumm). The peripheral smear demonstrated 85% blasts, and bone marrow aspiration revealed 82% blasts with reduced erythropoiesis and megakaryocytes, as shown in Figure 1. The biopsy also showed diffuse sheets of blast cells with comparable morphology.

**FIGURE 2:**
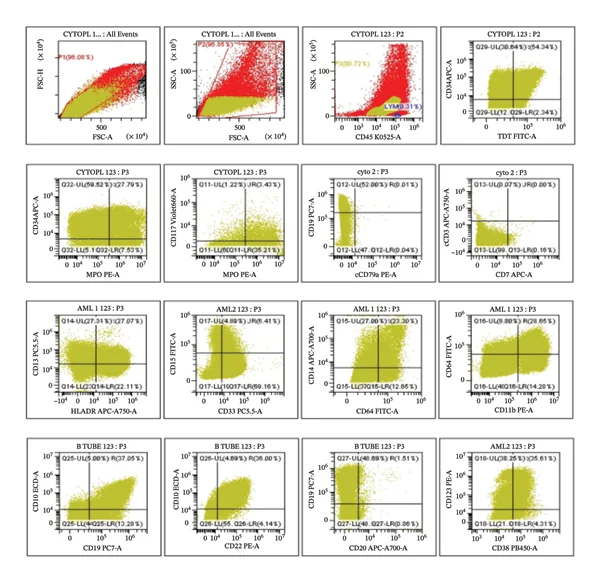
Immunophenotyping of the bone marrow aspirate demonstrated a distinct blast population gated on CD45 vs. SSC‐A. The blasts expressed B‐lineage markers, including CD19, CD22, and CD10, with coexpression of CD19 and CD10. Additionally, the blasts showed positivity for myeloid‐associated markers, including CD13, CD33, CD11b, CD14, CD64, CD123, and cytoplasmic myeloperoxidase (cytoMPO). The blasts also expressed precursor markers such as CD34, cytoplasmic TdT, and HLA‐DR.

Immunotyping was performed on a bone marrow aspiration sample demonstrating mixed phenotypic acute leukemia (B/Myeloid) as shown in Figure 2. Flow cytometry was performed on the Beckman Coulter DxFLEX Platform in bone marrow aspirate. The sample was processed, and cell preparation was performed using the stain‐lyse‐wash method. CD45 was used for gating strategy using CytExpert software. Diagnosis was given based on the WHO‐HAEM5 criteria for MPAL [4].

Blasts showed bright CD19 and CD10 coexpression, with dim CD13 and CD33, supporting B/Myeloid lineage assignment. Additional cytoplasmic MPO positivity supports the diagnosis of MPAL, B/Myeloid type, in accordance with WHO classification criteria [4].

Internal control populations, including residual lymphocytes and myeloid cells, showed appropriate staining patterns, confirming the specificity of antigen expression.

**FIGURE 3:**
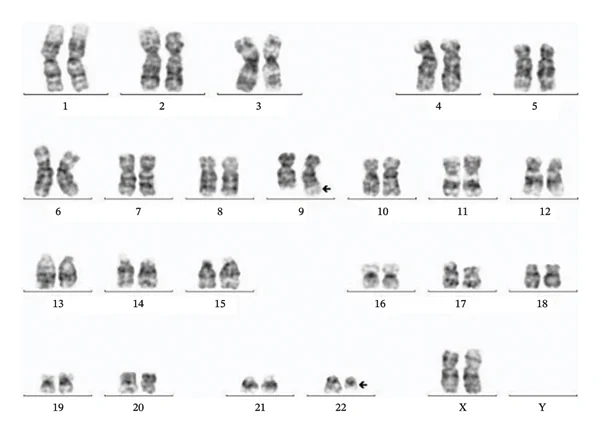
A conventional karyotype showed a translocation of the long arm of chromosomes 9 and 22, as shown by the arrow.

Karyotyping revealed a balanced reciprocal translocation between the long arms of chromosomes 9 and 22 between the regions q34 and q11.2, respectively, supporting the presence of the Philadelphia chromosome complement (Figure 3). Conventional cytogenetic analysis was performed using GTG banding following a 24‐h unstimulated culture. Five metaphases were counted (as there was a low mitotic index), analyzed, and karyotyped at a banding resolution of 300–550 bands per haploid set. The karyotype was 46, XX, t (9; 22) (q34; q11.2)[03]/48, idem, +mar1, +mar2[02], demonstrating a Philadelphia chromosome‐associated t (9; 22) translocation.

Also, RT‐PCR & Gel Electrophoresis for BCR/ABL1 Translocation Assay (Qualitative) was performed with EDTA Peripheral Blood as per the standard laboratory protocol. The test was directed at assessing transcripts for the Major, Minor, and Micro breakpoints corresponding to p210, p190, and p230. In the present case, the BCR–ABL1 major (p210) fusion transcript was detected.

She was then diagnosed as a distinctive entity of mixed phenotypic acute leukemia with t (9; 22). Our case was from a resource‐limited setting, where comprehensive genomics investigations were unavailable, and faced the diagnostic challenge. The diagnosis was made after a stepwise approach starting with morphological assessment, followed by immunophenotyping, conventional cytogenetic analysis, and targeted molecular testing. The patient received treatment with a BFM‐based ALL regimen with TKI [9] (including methotrexate, vincristine, dexamethasone, daunorubicin, and L‐asparaginase (Leunase) given in Phase I and cyclophosphamide, cytarabine, mercaptopurine, and methotrexate in Phase II). The patient showed significant improvement (postinduction B‐ALL MRD 0.01%) and a reduction in BCR–ABL1. The patient is on continuation therapy with multiagent chemotherapy along with TKI.

## 3. Discussion

Acute leukemia is of myeloid or lymphoid origin, which are distinct lineages. But a small percentage of acute leukemia is referred to as “ALAL” when it exhibits many lineages or no single lineage. This type of leukemia can be either “undifferentiated,” with no obvious indication of any lineage commitment, or an MPAL that combines the phenotype of two or more lineages. MPAL may consist of two separate populations of blasts, each expressing a different lineage‐specific antigen, or it may consist of a single population of blasts that expresses both particular myeloid and lymphoid antigens (biphenotype) [6]. Cytogenetic findings, based on conventional Karyotyping, demonstrated a translocation in the long arm of chromosomes 9 and 22 in all five metaphases analyzed. Among 5 metaphases, 3 of them showed t (9; 22) abnormality alone and 2 demonstrated the same abnormality with two additional marker chromosomes {46, XX, t (9; 22) (q34; q11.2)[03]/48, idem, +mar1, +mar2[02]. The presence of a subclone suggests the possibility of additional cytogenetic evolution within the leukemic clone although conventional Karyotyping alone determines the origin of the marker chromosomes [4, 10]. This observation is consistent with the concept that the acquisition of additional genetic or cytogenetic abnormalities may accompany progression or diversification of an established leukemic clone. However, this finding should be interpreted and cannot by itself establish a temporal sequence of clonal evolution because only five metaphases were available for analysis. Our patient had B and myeloid markers positive. MPAL patients usually exhibit anemia, recurrent infections, or bleeding tendencies, which are signs of acute leukemia. Furthermore, lymphadenopathy or hepatosplenomegaly may also exist [6]. In the current case, the patient presented with anemia, thrombocytopenia, and hyperleukocytosis; however, lymphadenopathy or hepatosplenomegaly was not present. The WHO classification states that the identification of distinct cytochemical and immunophenotypic markers suggestive of developmental lineages is necessary for the diagnosis of MPAL. The simultaneous expression of B‐lineage and myeloid markers in our patient, confirmed by flow cytometry and cytoplasmic MPO positivity, fulfilled the WHO 5th edition criteria for B/Myeloid MPAL [4, 11]. The diagnosis of MPAL presents several challenges. Accurate lineage assignment is critical, as misclassification can significantly impact treatment decisions. MPAL must be differentiated from BCR–ABL1–positive B‐ALL, AML with aberrant lymphoid markers, ALAL (NOS), and chronic myeloid leukemia in blast crisis. Multiple B‐cell markers can be expressed by a small percentage of AML with a translocation t (8, 21), a form of AML having a favorable prognosis. They are not, however, regarded as biphenotypic. Additionally, a range of lineages can be expressed by chronic myeloid leukemia in blast crisis. The latter is difficult to distinguish from the former since it resembles MPAL with the Philadelphia chromosome [2–4]. In our case, internal controls confirmed the specificity of antigen expression, and the intensity of lineage‐defining markers (bright CD19/CD10, dim myeloid markers) supported the diagnosis. A differential diagnosis table (Table 1) summarizes the distinguishing features. The differential diagnosis was established based on the WHO 5th edition classification of hematolymphoid tumors and relevant literature (Table 1), which summarizes the distinguishing features [4].

**TABLE 1:** Differential diagnosis of BCR–ABL1–positive MPAL in our case.

Differential diagnosis	Key features	In our case	Decision: inclusion/exclusion
B/Myeloid MPAL	Coexpression of B‐lineage markers (CD19, CD22, CD10) and myeloid markers (cytoMPO, CD13, CD33, CD11b, CD14, CD64, CD123)	Blasts expressed bright CD19, CD10, CD22, and coexpression of CD19/CD10; myeloid markers positive including cytoplasmic MPO; precursor markers CD34, TdT, HLA‐DR positive	Included: fulfills WHO HAEM5 criteria for B/Myeloid MPAL

BCR–ABL1–positive B‐ALL	B‐lineage markers only; MPO negative; BCR–ABL1 positive	Myeloid markers present (MPO, CD13, CD33, CD11b)	Excluded because blasts show definitive myeloid differentiation (cytoMPO positive)

AML with aberrant lymphoid markers	Strong myeloid markers (MPO), possible weak B‐lineage expression	B‐lineage markers strongly expressed (CD19, CD10, CD22)	Excluded: strong B‐lineage markers meet MPAL criteria

Chronic myeloid leukemia in blast crisis	BCR–ABL1 positive; history of chronic phase; peripheral blood features (splenomegaly, basophilia)	No documented chronic phase; peripheral counts not typical for CML blast crisis. No splenomegaly. No basophilia	Less likely: clinical history does not support prior CML

Acute leukemia of ambiguous lineage (NOS)	Does not meet clear lineage‐defining criteria	Both B and myeloid lineages clearly present	Excluded: WHO criteria for B/Myeloid MPAL fulfilled

The age of our patient was 39 years, which seems a little younger than previous studies. In a study by Neuendorff et al., the median age of AML with BCR–ABL and CML with blast crisis was found to be 64–66 years, whereas Matutes et al. found approximately 47 years [12, 13].

Comparable to Ph + ALL patients, Ph + MPAL patients, who were once thought to be indicators of bad outcomes, are now found to have a higher overall survival rate than those of other subtypes. Various studies considered that the improvements are associated with TKI usage, emphasizing that the treating physician should start a TKI as part of the Ph + disease treatment plan and check for BCR–ABL1 rearrangement as soon as feasible following diagnosis in all MPAL cases [4, 8, 13].

Therapeutically, BCR–ABL1–positive MPAL requires a tailored strategy combining ALL‐directed induction with tyrosine kinase inhibitor therapy. While long‐term outcomes remain limited, careful monitoring and individualized therapy are essential for optimizing prognosis [4, 13]. Our patient was also receiving an ALL‐based chemotherapy regimen along with a tyrosine kinase inhibitor.

In some cases, targeted therapy was found to be beneficial. Before the start of treatment, the patient should be evaluated for mutations in IDH1/2, BRAF, NOTCH1, and FLT3 that might be targeted with available or emerging agents, including tyrosine kinase and NOTCH1 inhibitors. For patients with corresponding mutations, the FDA has approved the drug Enasidenib (an IDH2 inhibitor) and Midostaurin (an FLT3 inhibitor). Children with a ZNF384r mutation and exhibiting increased FLT3 expression may also benefit from FLT3 inhibitor therapy [5, 14].

When compared to other forms of leukemia, MPAL has a poor prognosis because the mixed‐phenotype leukemic stem cells are chemoresistant due to slow replication; the blasts can change their phenotype in response to therapy; and some MPALs express high levels of multidrug resistance proteins. Other variables, such as increased patient age, unexplained molecular abnormalities, and induction therapy failure, may result in the disease’s progression [5, 8, 13, 14].

MPAL with BCR–ABL1 remains diagnostically challenging due to overlapping morphological and immunophenotypic features with AML and ALL. Misclassification may lead to suboptimal therapy, particularly if tyrosine kinase inhibitors are omitted. This report emphasizes the need for heightened awareness of MPAL with BCR–ABL1, representing a form of high‐risk acute leukemia with significant therapeutic implications, particularly in resource‐limited settings where diagnostic constraints may delay optimal management.

## 4. Conclusion

MPAL with BCR–ABL1 positivity is a rare entity, representing a diagnostic and therapeutic challenge. Resource‐limited settings pose additional challenges, including limited access to advanced immunophenotyping, cytogenetic, and molecular testing, which ultimately may delay optimal management. This case aims to increase awareness of MPAL, promote meticulous diagnostic evaluation, and provide insights into management in settings with limited laboratory resources.

**Nomenclature**
MPAL: Mixed phenotype acute leukemia
ALAL: Acute leukemias of ambiguous lineage
AUL: Acute undifferentiated leukemia
AML: Acute myeloid leukemia
ALL: Acute lymphoblastic leukemia

## Author Contributions

1. Sadichhya Poudel: preparation of the original draft, collection of images, history taking, and other details of the patient, and review.

2. Subidha Sharma: preparation of the original draft, collection of images, history taking, and other details of the patient, and review.

3. Shankar Kafle: data curation by acquiring and critically reviewing the selected articles and reviewing and editing to refine the manuscript.

4. Shovana Karki: conceptualization, investigation, and review of the case.

5. Anjan Shrestha: investigation, therapeutic management, and review of the case.

## Funding

No funds, grants, or other support were received.

## Disclosure

All authors have read and approved the final version of the manuscript. Dr Shankar Kafle had full access to all of the data in this study and takes complete responsibility for the integrity of the data and the accuracy of the data analysis. Authorea for a preprint that has previously been published (Poudel et al. 2026) [15].

## Ethics Statement

This study has been exempted from ethical approval by our institutional research committee.

## Consent

The patient provided written informed consent for publication and the use of case details, including images.

## Conflicts of Interest

The authors declare no conflicts of interest.

## Supporting Information

Additional supporting information can be found online in the Supporting Information section.

## Supporting information


**Supporting Information** MPAL case report CARE checklist.

## Data Availability

The authors will make all information and related data from this case report available upon request.
